# Interaction between the substrate and probe in liquid metal Ga: experimental and theoretical analysis†

**DOI:** 10.1039/d3ra04459a

**Published:** 2023-10-18

**Authors:** Ken-ichi Amano, Kentaro Tozawa, Maho Tomita, Riko Takagi, Rieko Iwayasu, Hiroshi Nakano, Makoto Murata, Yousuke Abe, Toru Utsunomiya, Hiroyuki Sugimura, Takashi Ichii

**Affiliations:** a Department of Applied Biological Chemistry, Faculty of Agriculture, Meijo University Nagoya 468-8502 Japan amanok@meijo-u.ac.jp; b Research Center for Computational Design of Advanced Functional Materials, National Institute of Advanced Industrial Science and Technology Tsukuba 305-8568 Japan; c Department of Materials Science and Engineering, Kyoto University Kyoto 606-8501 Japan ichii.takashi.2m@kyoto-u.ac.jp

## Abstract

Interaction between two bodies in a liquid metal is an important topic for development of metallic products with high performance. We conducted atomic force microscopy measurements and achieved the interaction between the substrate and the probe in liquid Ga of an opaque and highly viscous liquid. The interaction cannot be accessed with the normal atomic force microscopy, electron microscopy, and beam reflectometry. We performed a theoretical calculation using statistical mechanics of simple liquids by mixing an experimentally derived quantum effect. From both experiment and theory, we found an unusual behaviour in the interaction between the solvophobic substances, which has never been reported in water and ionic liquids. Shapes of the interaction curves between several solvophobic and solvophilic pairs in liquid Ga are also studied.

## Abstract 2

Interaction between two bodies in a liquid metal is an important topic for development of metallic products with high performance. We conducted atomic force microscopy measurements and theoretical calculations in liquid Ga.

## Graphical abstract

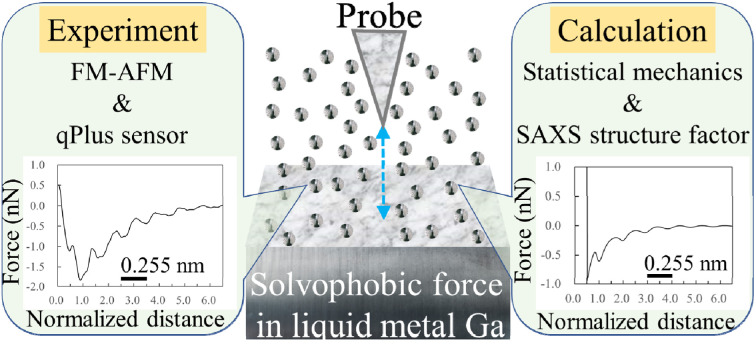

## 1 Introduction

Solid metals have high stiffness, strength, plasticity, and thermal and electrical conductivities. Improvements in these properties are essential for the development of metallic products. For example, high stiffness, strength, and plasticity are useful in artificial joints, reinforcing bars, and car bodies. In certain fields, nanoparticles are dispersed in solid metals to enhance the properties of these products. Hence, the dispersion stability of nanoparticles in a liquid metal is also an important topic.^1^

Liquid metals are used in batteries to avoid the growth of dendrites on electrodes,^2–4^ which leads to safe and long-life batteries. Nanoporous solid metals are some of the promising materials for electrodes, which can be fabricated in liquid metals.^5,6^ Liquid metals are also applied to cool computer chips,^7,8^ and to fabricate anisotropic piezoconductivity in piezoelectric elements.^9^ They are also used in thermal conductors (heat pipes) owing to their fluidity and high thermal conductivity. The heat pipe is applied to prevent permafrost melting^10^ in nuclear reactors^10,11^ and space reactor power systems.^11^ However, there are problems with aggregation and precipitation of impurities. By the way, natural^12^ and artificial^13^ diamonds which are used as jewels and product materials are made in a liquid metal with high pressure and temperature. Components of the diamond, carbon atoms, are dissolved in the liquid metal, when it is fabricated. Moreover, Gallium nitride (GaN), a famous crystal for blue light-emitting diodes (LEDs),^14,15^ can also be made in liquid metals.^16–18^ GaN crystals are important materials for fabrication of ultraviolet LEDs, high-power electronic devices, and high-frequency electronic devices. However, it is still difficult to make large crystals and control their qualities and properties. Considering these viewpoints and problems, the interaction between two substances in the liquid metal should be elucidated in more detail for development of the materials fabricated in the liquid metals.

In previous studies, the effective pair potentials between constituent atoms of liquid metals have been studied theoretically^19–23^ and experimentally.^24–26^ The theoretical studies^19–23^ have found a characteristic oscillation in the effective pair potential. Similarly, the characteristic oscillation has been experimentally determined using small-angle X-ray scattering (SAXS).^24–26^

Interactions between non-constituent particles (*i*.*e*., solutes and nanoparticles) in liquid metals have also been studied. The potential of mean force (PMF) between the SiC nanoparticles in a liquid metal has been thermodynamically predicted in a simple and targeted manner.^1^ In the thermodynamic method, the PMF values at the contact point and at the two slightly separated points were estimated. Starting from zero PMF at the sufficiently separated point, the curve of the PMF was drawn by a plausible smooth interpolation.^1^ Although it is not PMF between solutes, total pair correlation functions between metallic solutes, which can be used for calculation of the PMFs, have been calculated using an *ab initio* molecular dynamics (AIMD) simulation.^27^ The density profiles of a liquid metal near a substrate have also been calculated using the AIMD simulation.^28^ The AIMD simulation is theoretically more exact than the thermodynamic prediction. However, the system size, simulation time, and number of atoms in the AIMD simulation are generally not sufficient owing to its high computational costs. There are merits and demerits in the both thermodynamic and the AIMD methods.

In this study, we measured the interaction curves (force and PMF) between a probe and a substrate in liquid Ga using atomic force microscopy (AFM)^29^ to investigate true shapes of the force and PMF curves. Ichii *et al.*^29^ have developed AFM that can measure in a liquid metal of an opaque and highly viscous liquid. Ichii *et al.* have measured the topographic image of surfaces of the AuGa_2_ solid with atomic resolution in liquid Ga. Its step structure and crystal growth in liquid Ga have also been studied. Although AFM is a prevailing machine for the studies in liquids,^30^ to the best of our knowledge, this is the first time for AFM to measure force and PMF curves in a liquid metal.

Moreover, in this study, we theoretically studied the interactions using statistical mechanics of simple liquids^31–34^ to compare the interactions from the both theory and experiment. In our calculation, a quantum effect (Friedel oscillations)^23,35^ is implicitly included by introducing an effective pair potential between Ga atoms in liquid Ga measured by SAXS.^24^ Due to the theoretical simplicity, the system size and the number of atoms in our calculation are sufficiently large in contrast to the AIMD simulation.^27,28^ Shape of the PMF curve obtained from our calculation is sufficiently continuous and more reasonable, unlike the thermodynamic prediction.^1^

## 2 Methods

### 2.1 AFM experiment

The AFM experiments were performed under atmospheric conditions at room temperature using a frequency modulation (FM) technique, in which the forces acting on the sensor were detected as a resonance frequency shift (Δ*f*).^36^ A commercial AFM (JEOL Co.; JSPM-5200) with a Nanonis AFM control system (SPECS Zurich GmbH) was used, with some modifications. The AFM force sensor used in this study was a qPlus sensor fabricated using a commercial quartz tuning fork (STATEK Co. TFW-1165) and an electrochemically etched tungsten probe.^37,38^ The resonance frequency, amplitude, and spring constant of the tuning fork were 16 529 kHz, 30 pm, and 1884 N m^−1^, respectively. The probe was made of a tungsten wire with diameter 0.1 mm (Nilaco Co.) and was etched in a potassium hydroxide solution of 1.2 mol L^−1^. The qPlus sensor was mechanically vibrated by a lead zirconate titanate (PZT) piezoelectric actuator, and its deflection was electrically detected by a differential current amplifier embedded in the AFM head.^39^ The Δ*f* of the qPlus sensor was detected using a commercial FM demodulator (Kyoto Instruments, KI-2001) with some modifications,^40^ where the vibrating amplitude was kept constant by home-built feedback electronics.

We purchased liquid Ga from Nilaco Co. and its purity was 6N (≥99.9999%). Liquid Ga droplet (several microlitres) was deposited on a cleaved mica surface (Furuuchi Chemical Co.) in a dry chamber.^29^ Only the probe apex was inserted into the liquid Ga droplet. The Ga surface was oxidised instantaneously in the atmosphere. The oxide film formed on the Ga/air interface was penetrated by the probe, and the interface between the mica surface and liquid Ga was investigated using the probe.

An epitaxially-grown Au (111) film with a 100 nm thickness was prepared by vacuum evaporation (base pressure: ∼10^−5^ Pa, evaporation rate: ∼0.1 nm s^−1^) on a cleaved mica substrate. During the evaporation, the substrate temperature was kept at 723 K. A several μL of liquid Ga droplet was deposited on the Au film.^29^ The liquid Ga started to diffuse into the Au film shortly after the deposition, and Au–Ga alloy was formed. The AFM investigation on the Au–Ga alloy was carried out approximately 10 hours after the deposition of the liquid Ga, which was enough for the formation of the AuGa_2_ alloy crystals.^29^ The oxide film formed on the Ga/air interface was penetrated by the probe, and the interface between the alloy surface and liquid Ga was investigated using the probe.

The Δ*f*-distance curve was obtained by changing the tip-to-sample distance without the Δ*f* feedback at a constant surface position, and it was converted to the force–distance curve using the method developed by Sader and Jarvis.^41^ The two-dimensional (2D) force distribution was calculated from the 2D frequency shift (Δ*f*) distribution (Fig. S1a in ESI†). The 2D-Δ*f* distribution was obtained by repeating the Δ*f*-distance curve measurement without the Δ*f*-feedback with changing the lateral positions.

We note following two aspects. First, it is probable that the probe did not analyse the mica surface, but rather the Ga oxide film that covered the mica substrate. As described above, liquid Ga was dropped onto a mica surface in the dry chamber. The surface of the Ga droplet was already covered with the Ga oxide film,^29^ and it is likely that the oxide film covered the mica surface when the droplet was placed. It is deduced that the probe surface was also covered with the Ga oxide film. The second aspect that must be considered is the contact angle of the Ga droplet. The contact angle could not be measured accurately in air because of existence of the oxide film (not liquid) covering the Ga droplet/air interface. However, when the Ga droplet was placed on the substrate which was coated by the Ga oxide film, the wettability of the droplet on the substrate was poor in visual. Hence, the substrate surface was considered to be solvophobic. In a similar way, the probe surface was also considered to be solvophobic because of the Ga oxide film on the probe surface. This speculation can be supported by facts that the wettability of liquid Ga is poor for oxide materials such as quartz (Si_2_O_2_) and sapphire (Al_2_O_3_).^42^ Moreover, although it is a property of Galinstan (a mixture of Ga, In, and Sn), the contact angles of the mixed liquid on the oxide materials have been also reported to be larger than 90°.^42^

There is a concern that our AFM machine can only measure oxidized metal surfaces. However, it can be overcome by placing the machine in a glove box filled with Ar or N_2_ gas. Although we have not placed the machine in the glove box yet, we have already succeeded to measure the interaction curves on a surface of a non-oxidized substrate AuGa_2_.^29^ Its topographic image with atomic resolution^29^ has also been already obtained without the oxide film.

The reason why the lowest points in the 2D-Δ*f* distribution (Fig. S1a in ESI†) are not at the same level stems from the roughness of the substrate surface. This result reflects the fact that the substrate surface (mica) is covered with spontaneously formed gallium oxide film (GaO_*x*_), which is considered to be non-flat in comparison with a cleaved mica surface.

### 2.2 Integral equation theory

We employed integral equation theory (statistical mechanics of simple liquids), where we applied the Ornstein–Zernike (OZ) equation coupled with a hypernetted-chain (HNC).^31–34^ Using the OZ-HNC theory, the normalised number density distributions of liquid Ga near the substrate and the interactions (force and PMF) between a substrate and a probe in liquid Ga were calculated. The calculation temperature *T* was 333.15 K (60 °C) to avoid crystallisation. Because our calculation incorporates an effective pair potential between Ga cations in a sea of conduction electrons obtained from SAXS measured by Waseda *et al.*,^24^ our calculation implicitly contains quantum effects of conduction electrons in liquid Ga.

### 2.3 Pair potentials

**Fig. 1:**
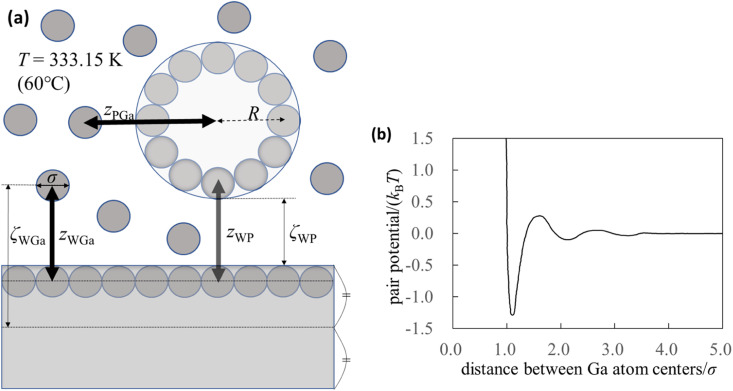
Pair potentials. (a) Schematic of the calculation system. Although the thickness of the substrate appears to be thin in the figure, it is ten times the diameter of the Ga atom. (b) Effective pair potential between the Ga cations in the sea of the conduction electrons (Waseda pair potential).^24^ The diameter of the Ga atom is represented by *σ* (=0.255 nm),^24^ and *k*_B_ and *T* are the Boltzmann constant and absolute temperature, respectively.

To understand the mechanism of interaction between the substrate and probe in liquid Ga, we used the integral equation theory. For the calculation, we prepared a simple system (Fig. 1a) and focused on the following four pair potentials.

The pair potential between the cations of the liquid Ga is explained below. Liquid Ga contains Ga cations and conduction electrons. It is difficult to treat the sufficient number of the conduction electrons in liquid Ga using the AIMD simulation. Hence, we used the effective pair potential between the Ga cations in the sea of the conduction electrons provided by Waseda *et al.*^24^ (Fig. 1b). The effect of conduction electrons is implicitly included in the pair potential. As shown in Fig. 1b, there are many oscillations caused by the Friedel oscillations.^23,35^ The reason for selecting the Waseda pair potential is as follows. There are several effective pair potentials between the constituent atoms in liquid metals.^19,20,43–46^ Some of pair potentials have been theoretically proposed, whereas the pair potentials obtained by Waseda *et al.* have been experimentally determined. Hence, the pair potentials obtained by Waseda *et al.* contains the real liquid metal conditions and properties. Hence, we selected the Waseda pair potential.

The pair potential between the substrate and the Ga cation in the sea of the conduction electrons is explained below. The pair potential between them (*U*_WGa_) was modelled using the following equation:^47,48^1
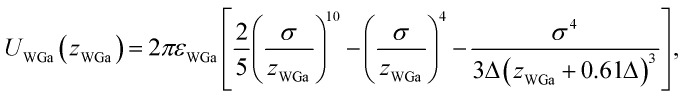
where *z*_WGa_, *σ*, and Δ are the distances between the centres of the substrate surface atom and Ga atom, the diameter of the Ga atom (0.255 nm),^24^ and 
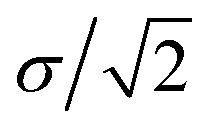
, respectively. Parameter *ε*_WGa_ represents the affinity between the substrate and the Ga cation, where we conveniently prepared both the weak (solvophobic) and strong (solvophilic) parameters (*ε*_WGa_ = 10^−22^ or 75 × 10^−22^ J). The pair potential between the substrate and Ga cation is shown in Fig. S4a in ESI.†

The pair potential between the probe and liquid Ga cation in the sea of the conduction electrons is explained below. The pair potential between them (*U*_PGa_) was modelled using the following equation:^47,48^2
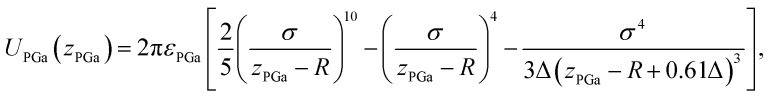
where *z*_PGa_ is the distance between the centres of the probe and Ga cation. Parameter *R* is the distance between the centres of the probe and probe surface atom. The parameter *ε*_PGa_ represents the affinity between the probe and Ga cations. Again, we conveniently prepared both weak (solvophobic) and strong (solvophilic) parameters (*ε*_PGa_ = 10^−22^ or 75 × 10^−22^ J). The pair potential between the probe and Ga cation is shown in Fig. S4b and S4c in ESI.†

The pair potential between the substrate and probe in a model continuum solvent of liquid Ga is explained below. The pair potential between them (*U*_WP_) was modelled as follows:^47,49^3
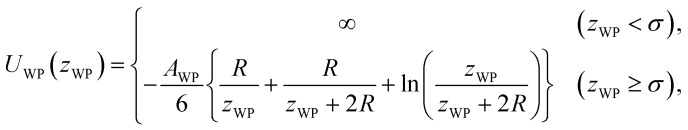
where *A*_WP_ and *z*_WP_ are the Hamaker constant between the substrate and probe in the continuum solvent of liquid Ga and the distance between the centres of the substrate surface atom and probe surface atom, respectively. The pair potential between the substrate and probe is shown in Fig. S4d in ESI.† From the AFM experiment, we considered that the surfaces of the substrate and the probe were composed of the gallium oxide films; hence, we set *A*_WP_ = 8.174 × 10^−19^ J. The value of *A*_WP_ was calculated using the following equation:^49^4
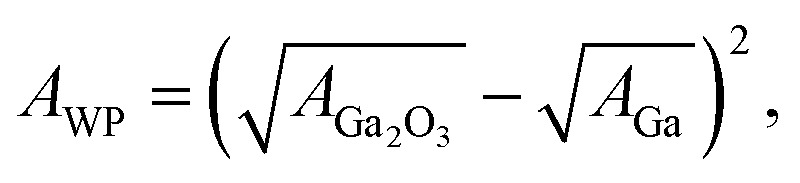
where *A*_Ga_2_O_3__ and *A*_Ga_ are the Hamaker constants of solid Ga_2_O_3_ and liquid Ga in vacuum, respectively. The Hamaker constant (*A*_Ga_2_O_3__ or *A*_Ga_) can be expressed using the corresponding surface tension, as follows:

5 *A* = 24π*l*^2^*γ*,

where *A*, *l*, and *γ* are the Hamaker constant of the substance of interest, the length of one side of a cube containing one molecule, the diameter of the molecule of interest, and the surface tension between solid Ga_2_O_3_ and vacuum (inert gas) or liquid Ga and vacuum (inert gas), respectively. Yunusa *et al.*^50^ reported the *apparent* surface tension between Ga_2_O_3_ and the gas as 591 mN m; hence, we applied the value for the surface tension. The apparent surface tension is not exactly the same as that between solid Ga_2_O_3_ and gas; however, we used this value because when liquid Ga_2_O_3_ is placed on solid Ga_2_O_3_, the contact angle should be close to *zero*. In this case, *γ*_sg_ (surface tension between the solid and gas) is nearly equal to *γ*_lg_ (surface tension between the liquid and gas). Therefore, a value of 591 mN m^−1^ was applied for *γ*_sg_. The surface tension between liquid Ga and gas was 695 mN m^−1^.^50^ Next, we explain *l* written in eqn (5) to obtain its value. The number density of amorphous Ga_2_O_3_ is 1.3 × 10^28^ m^−3^.^51^ (The number density of β-Ga_2_O_3_ has been reported^51^ to be 1.9 × 10^28^ m^−3^. However, the oxide film on liquid Ga is amorphous or poorly crystallised.^52^ Hence, we used the value of 1.3 × 10^28^ m^−3^. Although not shown, the qualitative conclusion of the present calculation does not change even when the value is 1.9 × 10^28^ m^−3^.) From the number density, the value of *l* for amorphous Ga_2_O_3_ was estimated to be 0.425 nm. In the integral equation theory, the temperature was set at 60 °C (*T* = 333.15 K) to converge the computation. Hence, we used the following number density of liquid Ga at 60 °C: 5.245 × 10^28^ m^−3^.^53^ Accordingly, the value of *l* for liquid Ga is 0.267 nm.

## 3 Results and discussion

### 3.1 AFM results

**Fig. 2:**
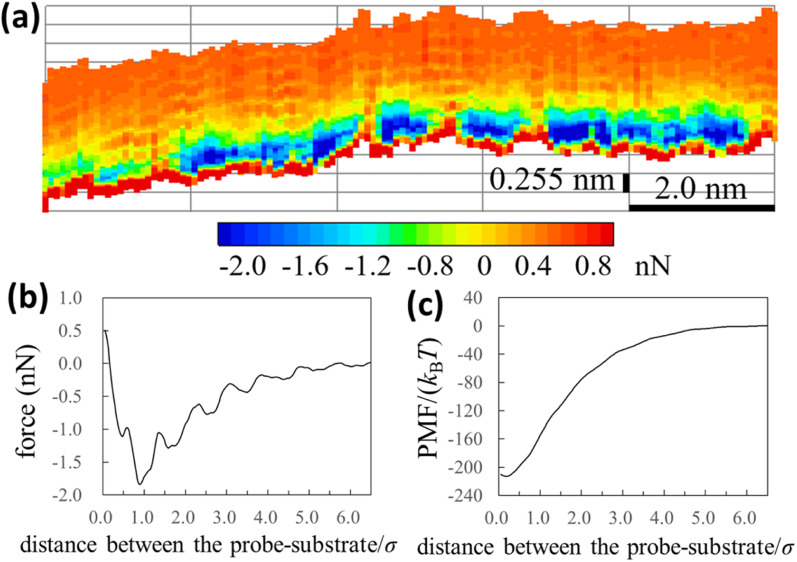
Solvophobic interactions in a liquid metal revealed by the AFM experiment. (a), (b), and (c) are 2D force distribution, force curve, and PMF curve between the gallium-oxide-coated substrate (solvophobic) and the gallium-oxide-coated probe (solvophobic) in liquid Ga, respectively.

We experimentally obtained the 2D force distribution between the substrate and probe in liquid Ga using AFM (Fig. 2a). To the best of our knowledge, this is the first revealed microscopic data in a liquid metal measured by AFM. Mica and tungsten^37,38^ were used as the substrate and probe, respectively. However, the mica and tungsten surfaces were coated with gallium oxide,^29^ and the surfaces are solvophobic due to the poor wettability. Hence, Fig. 2a represents the solvophobic interaction between the gallium oxide surfaces^51,52^ in liquid Ga. Fig. 2b is the force curve with a typical shape extracted from Fig. 2a. Fig. 2b depicts relatively many oscillations (six to seven observable oscillations) and strong attractive forces compared with the general AFM force curves in water.^31,54^ The oscillation length is approximately equal to the effective diameter of the Ga atom. By integrating the force curve, we obtained a PMF curve (Fig. 2c). A relatively deep PMF minimum was observed in the vicinity of the substrate surface. For reference, we included frequency shift data (original experimental data of Fig. 2) in the Fig. S1a and S2 in ESI.† 2D solvation structure roughly estimated from the 2D force distribution is also shown in Fig. S1b in ESI.†

**Fig. 3:**
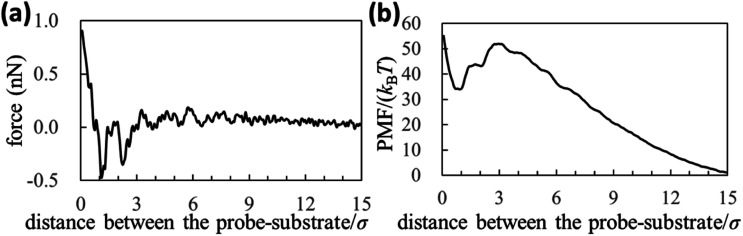
(a) Force and (b) PMF curves measured by our AFM on AuGa_2_ substrate surface. The experimental condition may correspond to a pair of the solvophilic substrate and the solvophobic probe.

**Fig. 4:**
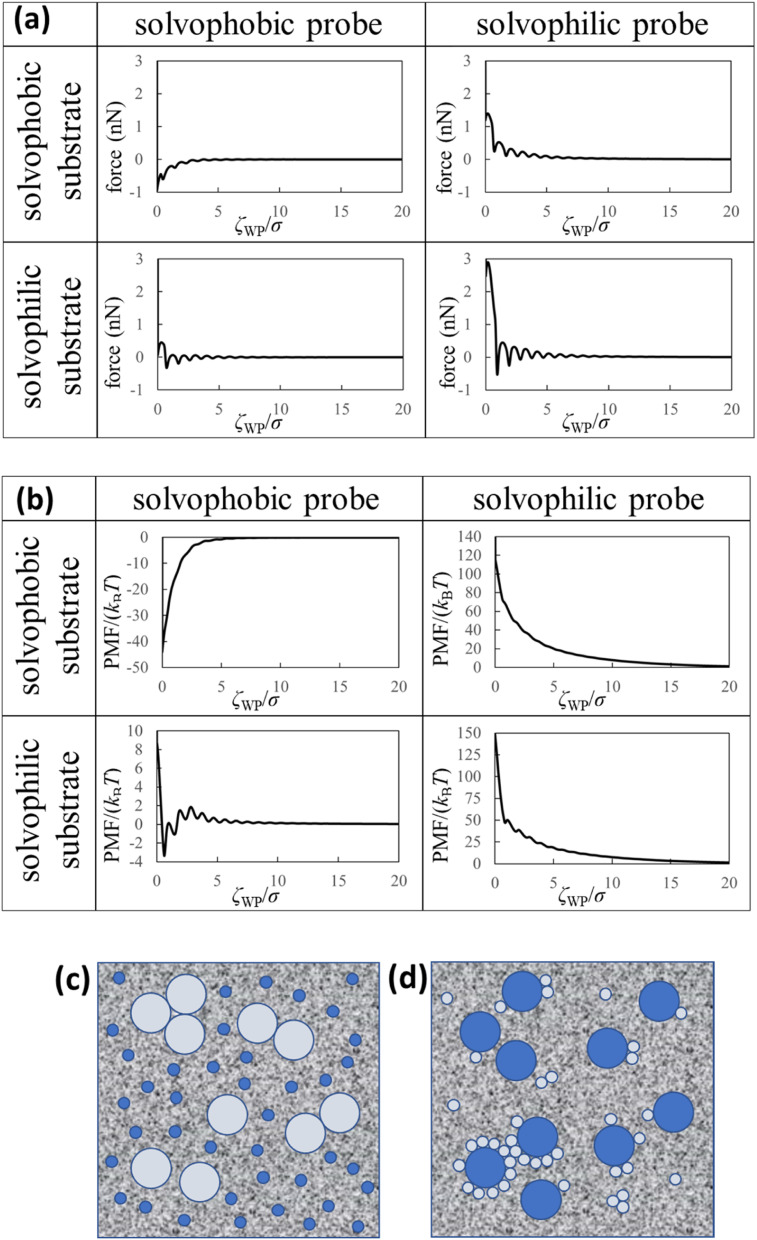
Theoretical results and dispersion/aggregation images (speculations) of the nanoparticles in the liquid Ga. (a) Force curves, (b) PMF curves between the solvophobic/solvophilic substrate and solvophobic/solvophilic probe in liquid Ga. The diameter of the probe is five times the diameter of the Ga atom (*i.e*. (2*R* + *σ*)/*σ* = 5). *ζ*_WP_ is the distance between the closest surfaces of the substrate and probe (Fig. 1a). (c) Schematic of the liquid metal containing the large solvophobic nanoparticles and the small solvophilic nanoparticles after adequate time. (d) Schematic of the liquid metal containing the large solvophilic nanoparticles and the small solvophobic nanoparticles after adequate time.

In Fig. 3a and b, we also show force and PMF curves measured by AFM on AuGa_2_ surface. For reference, we displayed frequency shift data (original experimental data of Fig. 3) in Fig. S3 in ESI.† It is considered that the probe surface was the gallium oxide and AuGa_2_ surface is not oxidized in liquid Ga.^29^ Hence, Fig. 3 shows the interactions between the solvophobic probe and the solvophilic substrate. In Fig. 3b, increase in PMF is observed from 15*σ* to 3*σ*, while decrease in PMF is observed from 3*σ* to 1*σ*. This behaviour (tendency) is related to a calculation result shown in Fig. 4b (bottom left). However, the oscillation lengths in Fig. 3a and b is about 1.5 times larger than *σ*. This is because the AuGa_2_ crystal surface plane was tilted with respect to the scanning direction of the probe.^29^ Although it is difficult to control the direction of the crystal surface, we consider that the oscillation length approaches to 1*σ* when the crystal surface and the scanning direction is vertical.

### 3.2 Theoretical results

We calculated the force curves, PMF curves, and density distributions of the Ga cations near surfaces of the substrate and probe using the affinity parameters *ε*_WGa_ (=10^−22^ or 75 × 10^−22^ J) and *ε*_PGa_ (=10^−22^ or 75 × 10^−22^ J). We defined values of the affinity parameters 10^−22^ J and 75 × 10^−22^ J as “solvophobic” and “solvophilic”, respectively. The solvophobic value was set so that minimum of the pair potential is almost nothing. The solvophilic parameter was set to almost the maximum value that can be handled in the integral equation theory. In the calculation, the diameter of the probe was five times the diameter of the Ga atom (*i.e*. (2*R* + *σ*)/*σ* = 5). Since the probe in our AFM experiment could measure the substrate surface with atomic resolution in liquid Ga, we estimated that the experimental probe was atomically sharp. For this reason, we prepared a probe five times the diameter of a Ga atom. We prepared the results when the diameter of the probe was ten times the diameter of the Ga atom, which are shown in Fig. S5 and S6 in ESI.†

Fig. 4a (upper left) demonstrates the force curve between the solvophobic substrate and solvophobic probe. A relatively strong attractive force exists because the substrate and probe are both solvophobic. In other words, the solvophobic attractive interaction exists also in liquid Ga. A comparison of Fig. 2b with Fig. 4a (upper left) shows that they are qualitatively similar (as a visual support, we prepared a figure for comparison in Fig. S7 in ESI†). There are relatively strong attractive forces and numerous oscillations. The amplitudes of the both force curves were also similar. We have considered that the surfaces of the substrate and probe in the experiment are solvophobic^40^ because they were coated with the gallium oxide.^51,52^ The consideration in the experiment was corroborated from a viewpoint of the shape consistency between the experiment and theory.

Fig. 4a (bottom right) shows the force curve between the solvophilic substrate and solvophilic probe. As shown in the figure, the force curve also contains many oscillations, which is similar to the other force curves. Unlike the force curve in Fig. 4a (upper left), there is a relatively strong repulsive force. This trend can be attributed to the surface affinities of the substrate and probe. Because both surfaces are solvophilic, the surfaces prefer to solvate with liquid Ga as much as possible. Hence, they are stable when they are separated rather than in contact.

Fig. 4a (upper right) shows the force curve between the solvophobic substrate and solvophilic probe. The force curve also exhibits many oscillations, and its shape is similar to that shown in Fig. 4a (bottom right). However, the repulsive force in the force curve is weaker than that in Fig. 4a (bottom right). Fig. 4a (bottom left) shows the force curve between the solvophilic substrate and solvophobic probe, which also exhibits many oscillations. The repulsive and attractive forces are observed in the force curve. Comparing the force curves presented in the upper right and the bottom left, it is found that the former contains mainly repulsive force while the latter contains both repulsive and attractive forces. That is, the results are different despite mere exchange of the surface affinities of the substrate and probe. We call this behaviour the “asymmetric property”. By the way, the results shown in Fig. 4a are qualitatively similar to those measured in an aqueous solution using AFM.^54^

Fig. 4b (upper left) demonstrates the PMF curve between the solvophobic substrate and solvophobic probe. There is a negative attractive potential because both the substrate and probe are coated with the gallium oxide^51,52^ (*i*.*e*., they are solvophobic surfaces^40^). We compared Fig. 2c with Fig. 4b (upper left) and found that they were qualitatively similar. Fig. 4b (bottom right) shows the PMF curve between the solvophilic substrate and solvophilic probe. Unlike the PMF curve in Fig. 4b (upper left), there is a positive repulsive potential. Fig. 4b (upper right) shows the PMF curve between the solvophobic substrate and solvophilic probe. The shape of the PMF curve is qualitatively similar to that shown in Fig. 4b (bottom right). However, the repulsive potential in Fig. 4b (upper right) is lower than that in Fig. 4b (bottom right). Fig. 4b (bottom left) shows the PMF curve between the solvophilic substrate and solvophobic probe. The whole shape is similar to the PMF curve experimentally obtained on the AuGa_2_ solvophilic surface in some degree (see Fig. 3b).

A comparison between Fig. 4b (upper right) and Fig. 4b (bottom left) shows that the shapes of both curves are definitely different from each other, despite mere exchange of the surface affinities of the substrate and probe. This is the asymmetric property in the PMF curves.

Here, we analogise *dispersion stability of the nanoparticles in liquid Ga* using Fig. 4a and b. For example, when the nanoparticles are solvophobic, their dispersion stability may be low because of attractive interactions. When the nanoparticles are solvophilic, their dispersion stability may be high owing to repulsive interactions among them. Interestingly, the solvophilic repulsive interaction arises at relatively long distances, the result of which cannot be obtained from thermodynamic theory.^1^ This is an advantage of our statistical mechanics theory over the thermodynamic theory.

We consider two types of nanoparticles in liquid Ga (see Fig. 4c). One is a relatively large nanoparticle with a solvophobic surface, and the other is a relatively small nanoparticle with a solvophilic surface. From Fig. 4a and b (upper right), the repulsive interactions between the large and small nanoparticles can be analogised. Therefore, when the two types of the nanoparticles are immersed in liquid Ga, probability of forming aggregates consisting of the large solvophobic nanoparticles and the small solvophilic nanoparticles is speculated to be small. However, the large nanoparticles with solvophobic surfaces may form aggregates. On the other hand, the small nanoparticles with solvophilic surfaces may stably dispersed in liquid Ga.

Next, we consider the following situation: one is a relatively large nanoparticle with a solvophilic surface and the other is a relatively small nanoparticle with a solvophobic surface (see Fig. 4d). From the theoretical results of Fig. 4a and b (bottom left), the attractive interactions between the large and small nanoparticles can be analogised (analogy drawn from Fig. 3 is not used here). Hence, when these particles are immersed in liquid Ga, they might aggregate after a sufficient amount of time. However, the large solvophilic nanoparticles themselves may dispersed in liquid Ga. The small solvophobic nanoparticles themselves may form aggregates in liquid Ga.

As shown in Fig. 2 and 4, we experimentally and theoretically observed the solvophobic interaction in liquid Ga. We found the relatively many oscillations in the interaction curves. For example, it has been reported that the hydrophobic interaction curves measured^54^ and calculated^55,56^ in water have relatively a few oscillations. Such property has been also reported in ionic liquids experimentally^57^ and theoretically.^58–60^ Hence, the solvophobic interaction in liquid Ga is distinct from the previously known solvophobic interactions. It is considered that the peculiar behaviour in liquid Ga stems from a quantum effect arising from the conduction electrons (*i.e.*, Friedel oscillations).^23,35^ This is because, presence of the conduction electrons is the major difference against water and ionic liquids. In fact, the peculiar behaviour disappeared when the effective pair potential between Ga cations (Fig. 1b) was replaced with a rigid pair potential (not shown). The results and more discussion will be presented in our future paper.

**Fig. 5:**
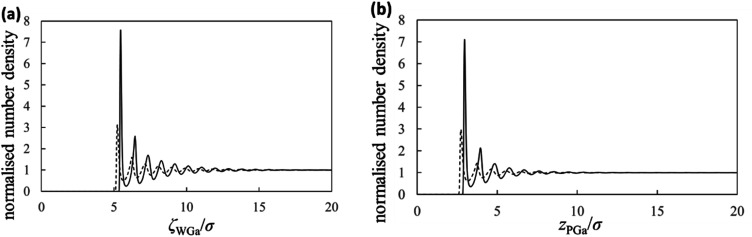
Solvation structures. Normalised number densities of the liquid Ga near (a) the substrate surface and (b) the probe surface. The solid and dashed curves are the normalised number densities near the surfaces of the strong and the weak affinities, respectively.

Next, we show the normalised number density distributions of the Ga atoms near the substrate (*g*_WGa_) and probe (*g*_PGa_) surfaces obtained from the integral equation theory in Fig. 5. The solid and dashed curves are the normalised number densities near the surfaces of the strong (solvophilic) and the weak (solvophobic) affinities, respectively. The first peaks in the normalized number densities near the solvophobic surfaces are clearly larger than 1 despite of their solvophobicity. The both solid and dashed curves show many oscillations. Their oscillation lengths are almost equal to the diameter of the Ga atom. Because there is a large difference in the solvation affinities of the surfaces, the heights of the first peaks exhibit the large difference. Although there is the large height difference, shapes of the both *g*_WGa_ curves are qualitatively similar. On the other hand, shapes of the force curves in Fig. 4a are apparently dissimilar. This trend exists also in the PMF curves in Fig. 4b. These trends in Fig. 4a and b are interesting, because the normalized number densities (original data for the force and PMF curves) on the solvophilic and solvophobic surfaces have similar shapes unlike the force and PMF curves.

## 4 Conclusions

The experimental and theoretical interaction curves were obtained and compared, and the qualitative agreement between them was confirmed. Many oscillations and strong attractive/repulsive interactions were observed in the interaction curves. From the experimental and theoretical results, we conclude that the solvophobic attraction in liquid Ga is unusually strong and containing many oscillations compared with that in water and ionic liquids. It is assumed that the unusual behaviour in the liquid metal originates from the quantum effect of the conduction electrons (Friedel oscillations).^23,35^ Force and PMF curves between the solvophobic probe and the solvophilic substrate were also measured with our AFM, shapes of which were somewhat similar to those obtained from the integral equation theory. From the integral equation theory, we found an asymmetric property in the shapes of the interaction curves. We believe that these results are practical for controlling the dispersion stability and self-assembly of nanoparticles in liquid metals. Our AFM and the findings may be helpful for development of Derjaguin–Landau–Verwey–Overbeek (DLVO) like interaction theory of easy-to-use in a liquid metal. As is well known, the DLVO theory that describes interactions between two bodies in electrolyte aqueous solutions has been greatly supported the developments of various products (*e*.*g*., pharmaceutical, cosmetic, and coating products). Hence, study of the interaction theory for a liquid metal may be also helpful for development of certain metallic products.

## Author contributions

All of the authors contributed to preparation of the manuscript. T. I. proposed the experimental direction of this research and K. A. and H. N. proposed the theoretical direction of this research. I. T. developed the FM-AFM. I. T., M. M., and Y. A. measured the force curve. K. A., K. T., M. T., and R. T. developed the calculation program. K. A., K. T., M. T., R. T., and R. I. performed the computation and prepared the figures. K. A., T. I., T. U., and H. S. investigated the experimental background of the liquid metal. K. A., K. T., M. T., R. T., R. I., and H. N. investigated the theoretical background of the liquid metal.

## Conflicts of interest

There are no conflicts of interest to declare.

## Supplementary Material

RA-013-D3RA04459A-s001
