# Supplementary material for: Interaction between the substrate and probe in liquid metal Ga: experimental and theoretical analysis
Source: RSC Adv. 2023 Oct 18;13(44):30615–24. doi: 10.1039/d3ra04459a (PMC10582826; doi:10.1039/d3ra04459a)
Supplement: RA-013-D3RA04459A-s001 [file RA-013-D3RA04459A-s001.pdf]

Electronic Supplementary Material (ESI) for RSC Advances.
This journal is © The Royal Society of Chemistry 2023

Electronic Supplementary Information

Interaction between substrate and probe in liquid metal Ga: Experimental and theoretical analysis

Authors: Ken-ichi Amano,[1]* Kentaro Tozawa,[1] Maho Tomita,[1] Riko Takagi,[1] Rieko Iwayasu,[1] Hiroshi Nakano,[2] Makoto Murata,[3] Yousuke Abe,[3] Toru Utsunomiya,[3] Hiroyuki Sugimura,[3] and Takashi Ichii,[3]*

[1]Faculty of Agriculture, Meijo University, Nagoya, Aichi 468-8502, Japan

[2]Research Center for Computational Design of Advanced Functional Materials, National Institute of Advanced Industrial Science and Technology, Tsukuba, 305-8568, Japan

[3]Department of Materials Science and Engineering, Kyoto University, Kyoto 606-8501, Japan

*e-mail: amanok@meijo-u.ac.jp; ichii.takashi.2m@kyoto-u.ac.jp

Figs. S1a and S1b shows a two-dimensional (2D) frequency shift distribution and an approximate 2D solvation structure (approximate 2D total correlation function), respectively. The 2D solvation structure was calculated from the 2D frequency shift distribution. First, we calculated the 2D force distribution (Fig. 2a) by using the Sader-Jarvis equation:[1]

$$F(z) = 2k\int_z^{\infty}\left(\left(1 + \frac{a^{1/2}}{8\sqrt{\pi(t-z)}}\right)\Omega(t) - \frac{a^{3/2}}{\sqrt{2(t-z)}}\frac{d\Omega(t)}{dt}\right)dt, \qquad (S1)$$

where F is the force between the probe and the substrate, k is the spring constant of the cantilever, a is the amplitude of the cantilever's oscillation, z is distance between the closest surfaces of the substrate and the probe, and t is the variable of the integration with respect to z. Here, $\Omega(t) = \Delta\omega(t)/\omega_{res}$, where $\Delta\omega(t)$ is the change in resonant frequency and ω_{res} is its unperturbed resonant frequency. The approximate 2D solvation structure (a function of q) is shown in Fig. S1b. We prepared the function q which is proportional to the total correlation function h (*i.e.*, $q \propto h$). The function q was calculated by using an equation $q = -\partial F/\partial z$ which is an approximate and simple relationship between the structure and the force.[2] Combining the Sader-Jarvis equation[1] and the relationship,[2] the equation for the 2D solvation structure is expressed as

$$h(z) \propto q(z) = 2k\left\{\Omega(t) - \int_z^{\infty}\left(\frac{a^{1/2}}{8\sqrt{\pi(t-z)}}\frac{d\Omega(t)}{dt} - \frac{a^{3/2}}{\sqrt{2(t-z)}}\frac{d^2\Omega(t)}{dt^2}\right)dt\right\}. \qquad (S2)$$

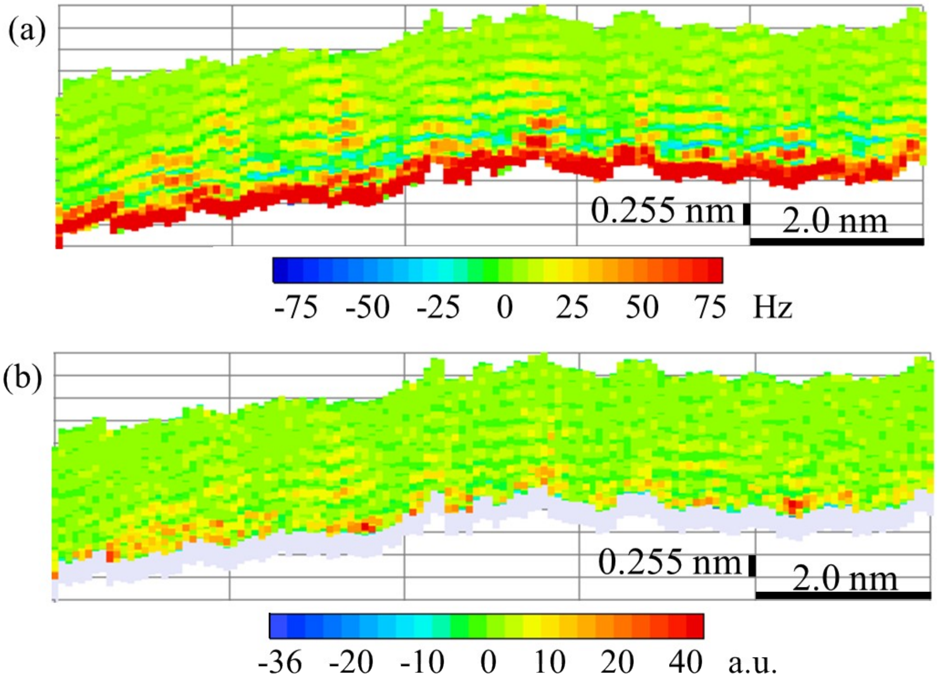

Fig. S1 (a) 2D frequency shift distribution. It is 256 frequency shift curves between substrate and probe in liquid Ga measured by the AFM experiment, where the substrate (mica) and the probe (tungsten) are covered with gallium oxide film.[3–5] (b) Approximate 2D solvation structure calculated from (a). The gray area is the substrate area which represents the approximate excluded area for the centers of Ga atoms.[6–9] The reason why the lowest points in the 2D frequency shift and the 2D solvation structure are not at the same level stems from the roughness of the substrate surface. This result reflects the fact that the substrate surface is covered with spontaneously formed gallium oxide film (GaO_x), which is considered to be non-flat compared with that of a cleaved mica surface.

By the way, eqn (S2) can be simplified depending on the cantilever's amplitude a. When a is sufficiently small, it can be expressed as

$$h(z) \propto q(z) = 2k\Omega(t). \qquad (S3)$$

This greatly simple approximate equation indicates that the frequency shift of the raw AFM data is directly proportional to the solvation structure (total correlation function). We think eqn (S3) is helpful for study of the solvation structure with AFM. We note that eqn (S3) cannot be applied to a situation that the probe and the substrate are directly interacting by their steric repulsive force.

In what follows, we show additional figures. Figs. S2 and S3 are additional AFM measurement results. Figs. S4-S6 are additional theoretical results. Fig. S7 shows experimental and theoretical solvophobic force curves for comparison. For more details, please check the figure captions.

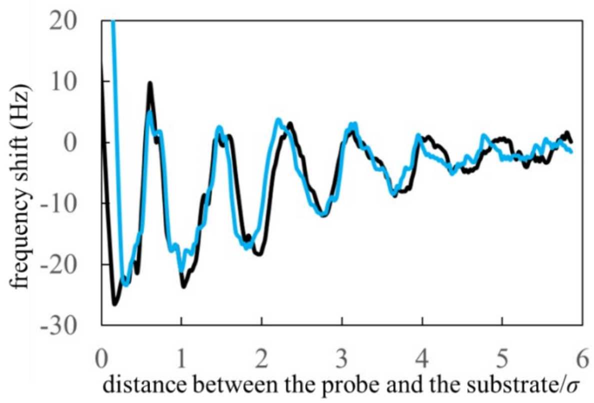

Fig. S2 Frequency shift curves measured by our AFM. Black and blue curves represent the approach and retract curves. Substrate (mica) and probe (tungsten) are covered with gallium

oxide film.[3–5]

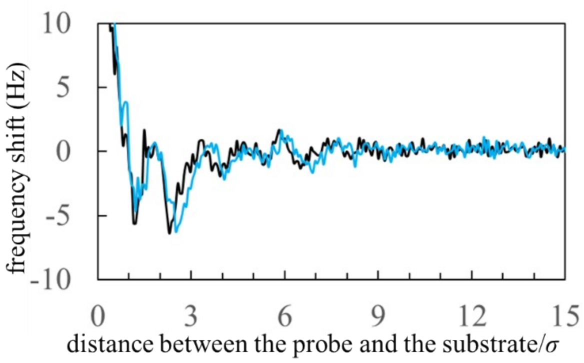

Fig. S3 Frequency shift curves measured by our AFM. Black and blue curves represent the approach and retract curves. Probe (tungsten) is covered with gallium oxide film.[3–5] Substrate surface is $AuGa_2$. The experimental condition may correspond to a pair of the solvophobic probe and the solvophilic substrate.

Models of the pair potentials between the substrate and Ga atom, the probe and Ga atom, and the substrate and probe were expressed by eqns. (1), (2), and (3). The pair potentials calculated from eqns. (1), (2), and (3) are shown in Figs. S4a, S4b-S4c, and S4d, respectively.

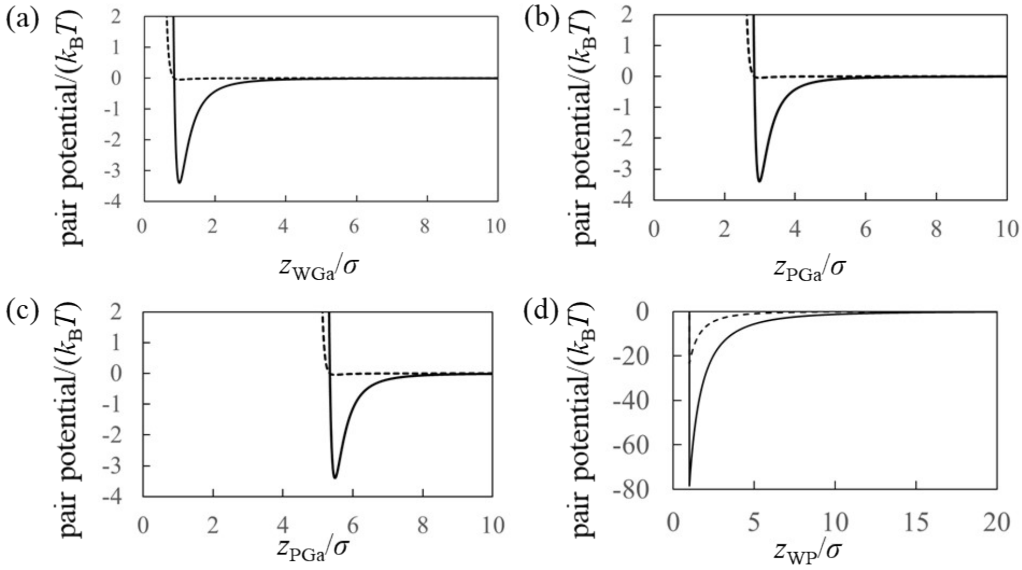

Fig. S4 (a) Solid and dashed curves are the pair potentials between the substrate and Ga atom when the affinity parameters are strong and weak, respectively. (b), (c) Solid and dashed curves represent the pair potentials between the probe and Ga atom when the affinity parameters are strong and weak. In (b), the diameter of the probe is five times that of the Ga atom (*i.e.*, $(2R + \sigma)/\sigma = 5$). In (c), the diameter of the probe is ten times that of the Ga atom (*i.e.*, $(2R + \sigma)/\sigma = 10$). (d) The solid and dashed curves denote the pair potentials between the substrate and probe when the probe sizes are ten and five times that of the liquid Ga atom, respectively. k_B and T are the Boltzmann constant and absolute temperature, respectively.

We show the force curves, PMF curves, and density distributions of the Ga atoms near a probe, where the diameter of the probe is ten times the diameter of the Ga atom (*i.e.*, $(2R + \sigma)/\sigma = 10$). The force curves, PMF curves, and density distributions of the Ga atoms near the probe calculated from the OZ-HNC theory are shown in Figs. S5 and S6. Following affinity parameters $\varepsilon_{\mathrm{WGa}}$ (= 10^{-22} or 75×10^{-22} J) and $\varepsilon_{\mathrm{PGa}}$ (= 10^{-22} or 75×10^{-22} J) are used. Here, we define values of the affinity parameters "10^{-22} J" and "5×10^{-21} J" as "solvophobic" and "solvophilic", respectively.

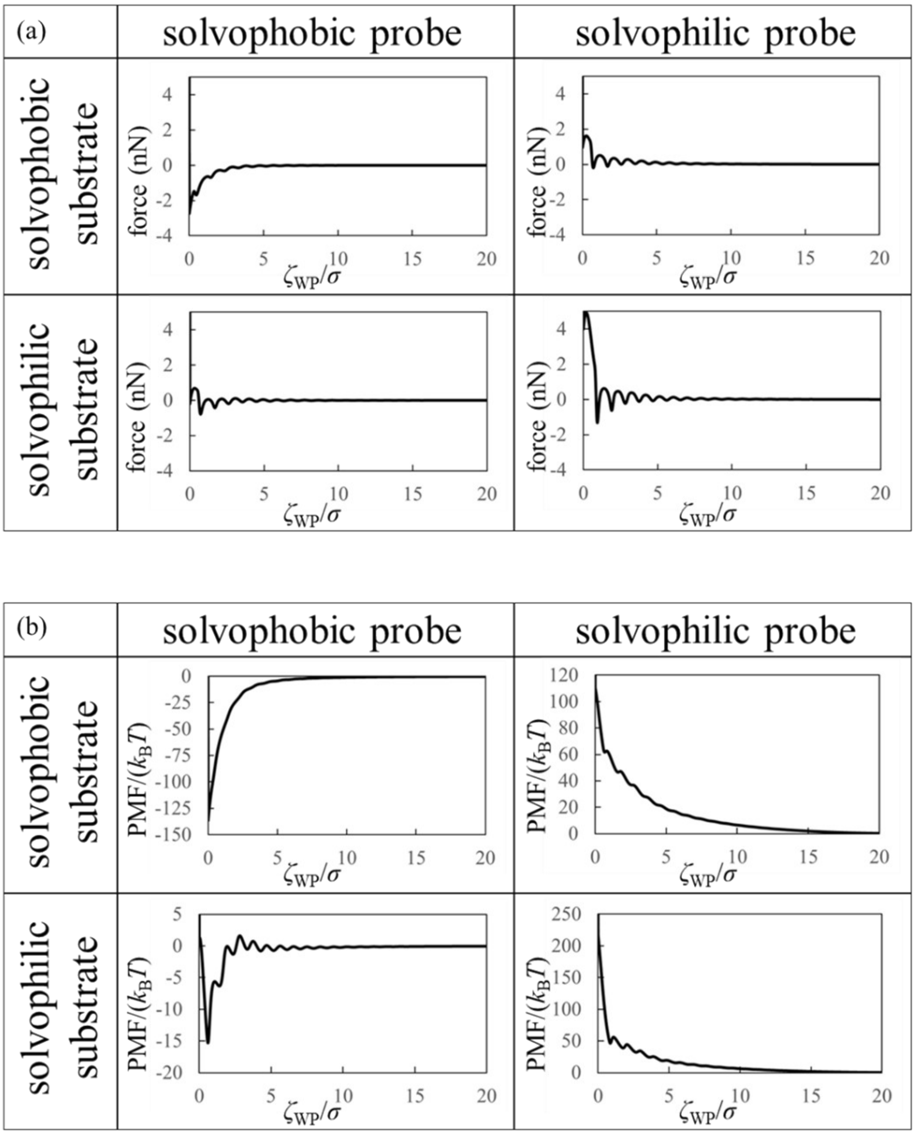

Fig. S5 (a) Force curves and (b) PMF curves between the solvophobic/solvophilic substrate and the solvophobic/solvophilic probe in the liquid Ga when the probe size is ten times that of the liquid Ga atom. ζ_{WP} is the distance between the closest surfaces of the substrate and the probe (see Fig. 1a).

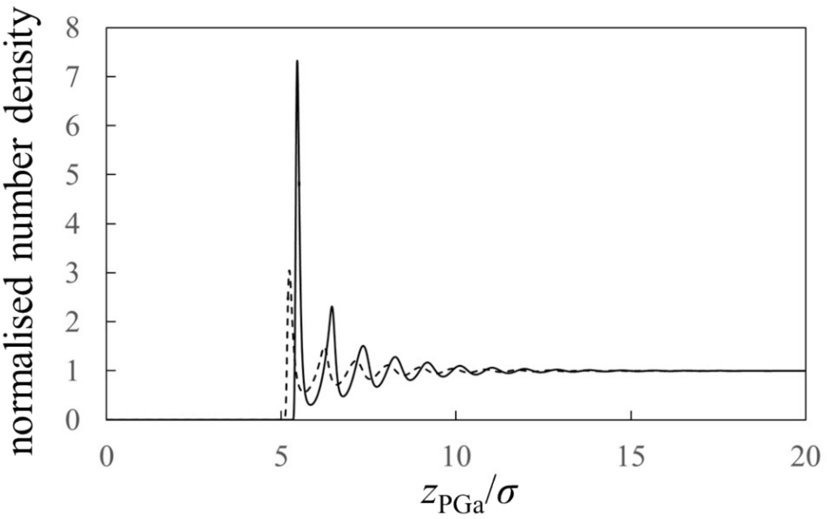

Fig. S6 Normalised number densities of the liquid Ga near the probe surface when the probe size is ten times that of the liquid Ga atom. The solid and dashed curves denote the normalised number densities on the solvophilic and solvophobic surfaces, respectively. z_{PGa} is the distance between the centres of the probe and the Ga atom (see Fig. 1a).

For comparison, we show the solvophobic force curves measured and calculated in liquid Ga in Fig. S7. As shown in Fig. S7, shapes of these force curves are qualitatively similar.

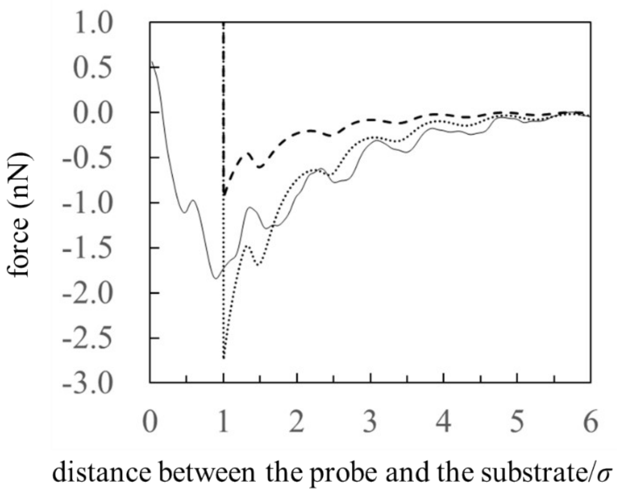

Fig. S7 Force curves between the solvophobic probe and substrate. The solid curve is the force curve measured by our AFM, which is the same as that in Fig. 2b. The dashed curve is the force curves calculated by the integral equation theory where the diameter of the probe is five times larger than that that of Ga atom, which is the same as that in Fig. 4a (upper-left). The dotted curve is the force curves calculated by the integral equation theory where the diameter of the probe is ten times larger than that that of Ga atom, which is the same as that in Fig. S5a (upper-left). For visual purposes, the dashed and dotted curves are shifted to the right by a unit length.

■ REFERENCES

1 J. E. Sader and S. P. Jarvis, *Appl Phys Lett*, 2004, **84**, 1801–1803.

2 K. Hashimoto, K. I. Amano, N. Nishi, H. Onishi and T. Sakka, *Journal of Chemical Physics*, 2021, **154**, 1564702.

3 M. J. Regan, H. Tostmann, P. S. Pershan, O. M. Magnussen, E. Dimasi, B. M. Ocko and M. Deutsch, 1997, **55**, 786–790.

4 E. Kobayashi, M. Boccard, Q. Jeangros, N. Rodkey, D. Vresilovic, A. Hessler-Wyser, M. Döbeli, D. Franta, S. De Wolf, M. Morales-Masis and C. Ballif, *Journal of Vacuum Science & Technology A: Vacuum, Surfaces, and Films*, 2018, **36**, 021518.

5 T. Ichii, M. Murata, T. Utsunomiya and H. Sugimura, *Journal of Physical Chemistry C*, 2021, **125**, 26201–26207.

6 K. Amano and O. Takahashi, *Physica A: Statistical Mechanics and its Applications*, 2015, **425**, 79–89.

7 K. I. Amano, E. Tanaka, K. Kobayashi, H. Onishi, N. Nishi and T. Sakka, *Surface Science*, 2015, **641**, 242–246.

8 K. I. Amano, Y. Liang, K. Miyazawa, K. Kobayashi, K. Hashimoto, K. Fukami, N. Nishi, T. Sakka, H. Onishi and T. Fukuma, *Physical Chemistry Chemical Physics*, 2016, **18**, 15534–15544.

9 K. Amano, T. Ishihara, K. Hashimoto, N. Ishida, K. Fukami, N. Nishi and T. Sakka, *Journal of Physical Chemistry B*, 2018, **122**, 4592−4599.
